# Minimum spectral resolution for continuous-wave hyperspectral near-infrared tissue spectroscopy

**DOI:** 10.1117/1.JBO.30.4.047002

**Published:** 2025-04-24

**Authors:** Ann Ping, Redwan Haque, Natalie C. Li, Rasa Eskandari, Mamadou Diop

**Affiliations:** a University of Toronto, Department of Mechanical and Industrial Engineering, Toronto, Ontario, Canada; b Western University, Department of Medical Biophysics, London, Ontario, Canada; c Western University, School of Biomedical Engineering, London, Ontario, Canada; d Imaging Program, Lawson Research Institute, London, Ontario, Canada

**Keywords:** near-infrared spectroscopy, hyperspectral, broadband near-infrared spectroscopy, continuous-wave, spectral resolution, tissue oximetry

## Abstract

**Significance:**

Continuous-wave hyperspectral near-infrared spectroscopy (h-NIRS) is a reliable and cost-effective technique for noninvasive monitoring of tissue blood content and oxygenation at the point-of-care; however, most h-NIRS devices are based on expensive custom-built spectrometers. For widespread adoption, low-cost, miniature, off-the-shelf spectrometers are needed. To guide the development of such spectrometers, a standard for spectral resolution must first be defined.

**Aim:**

We aim to identify the minimum spectral resolution needed for h-NIRS devices to accurately measure oxy- and deoxy-hemoglobin (HbO and Hb) concentrations in tissue.

**Approach:**

h-NIRS measurements were acquired from fully oxygenated and deoxygenated blood-lipid phantoms at 13 spectral resolutions. Data for other oxygenation levels were simulated using NIRFAST. HbO and Hb concentrations were estimated at each resolution and compared with the ground truth hemoglobin concentration.

**Results:**

The concentration of Hb was estimated with high accuracy for resolutions up to 10 nm, whereas HbO estimates were more variable. For both chromophores, the accuracy of the estimation gradually decreased with resolutions beyond 10 nm.

**Conclusions:**

Spectral resolutions up to 10 nm can be used for h-NIRS without compromising the accuracy of estimating tissue blood content and oxygenation.

## 1 Introduction

Near-infrared spectroscopy (NIRS) is an effective technique for noninvasive monitoring of cerebral hemodynamics, especially in neonates due to their thinner scalp and skull.^1^^–^^5^ To quantify tissue blood content and oxygenation with NIRS, the effects of light scattering must be separated from those of absorption. This can be achieved with continuous-wave hyperspectral NIRS (h-NIRS), which estimates the concentrations of oxy- and deoxy-hemoglobin (HbO and Hb) from light attenuation at dozens of wavelengths rather than just a discrete few. In this approach, the contribution of scattering is dampened, and absolute absorption is estimated through spectral differentiation.^6^^–^^15^ Moreover, h-NIRS uses simple instrumentation and computationally efficient algorithms, unlike frequency-domain and time-resolved NIRS, making it a promising approach for wider clinical adoption.^16^^–^^19^ Currently, most h-NIRS devices for *in vivo* tissue monitoring are based on custom-built spectrometers^12^^,^^20^^–^^26^; however, the development of low-cost, miniature, off-the-shelf spectrometers would offer several advantages, including greater accessibility, smaller footprint, and portability for point-of-care applications.

A key characteristic of an optical spectrometer is the width of its entrance slit. A narrower slit provides superior spectral resolution, thereby improving the spectral accuracy of the measurements^27^; however, a narrow slit also limits light collection. This is important because efficient light collection is critical in tissue spectroscopy because only a small fraction of the injected photons emerge from the highly attenuating tissue.^10^ Thus, an effective slit width must balance the inherent trade-off between spectral resolution and light throughput [i.e., signal-to-noise ratio (SNR)]. The negative impact of a narrow slit width on SNR can be partially compensated for by increasing the integration time, but long integration times slow data acquisition, hindering real-time applications.

Furthermore, slit width and spectral resolution are not standardized across the literature, with most units prioritizing fine spectral resolution (0.3 to 5.5 nm).^10^^,^^12^^,^^20^^,^^23^^–^^26^^,^^28^ Interestingly, as the spectral features of HbO and Hb absorption are broad in the near-infrared region, their concentrations could potentially be quantified from measurements taken at coarser resolutions with wider slit widths, allowing for improved light collection and higher SNR. The aim of this work is to determine the minimum spectral resolution needed to accurately estimate tissue blood content and oxygenation by analyzing (1) attenuation measurements at various resolutions from blood-lipid tissue-mimicking phantoms and (2) simulated tissue light attenuation at the same resolutions. Identifying this minimum resolution could guide the design of future low-cost tissue spectrometers.

## 2 Materials and Methods

### 2.1 Instrumentation

A laboratory-grade spectrometer with a variable entrance slit (Sciencetech Inc., London, Ontario, Canada), a broadband halogen light source (Ocean Insight HL-2000-HP, Orlando, Florida, United States), and a 785-nm long-coherence laser (CrystaLaser, Reno, Nevada, United States) were used in this study. The halogen lamp was used to acquire hyperspectral NIRS measurements from tissue-mimicking phantoms, whereas the laser was employed to quantify detection-induced spectral broadening—due to degradation of the resolution at widening slit widths—for the simulations. The detector of the spectrometer was a two-dimensional charge-coupled device (CCD) array (S16001-1007S, Hamamatsu, Japan; 1024×122  pixels, pixel size of $24\times 24\;{\mu m}^{2}$, detection range 200 to 1100 nm). The full width at half maximum (FWHM) of the narrow-linewidth 785-nm laser was used to quantify the linear relationship between the slit width and the resolution of the spectrometer. The resolutions tested in this study are listed in Table 1.

**Table 1:** Spectral resolutions at the 13 slit widths tested in the study.

Slit width (μm)	50	100	200	500	1000	1500	2000	2500	3000	3500	4000	4500	5000
Resolution (nm)	0.9	1.1	1.5	2.6	4.5	6.4	8.4	10.3	12.2	14.1	16.0	18.0	19.9

### 2.2 Phantom Experiments

**Fig. 1:**
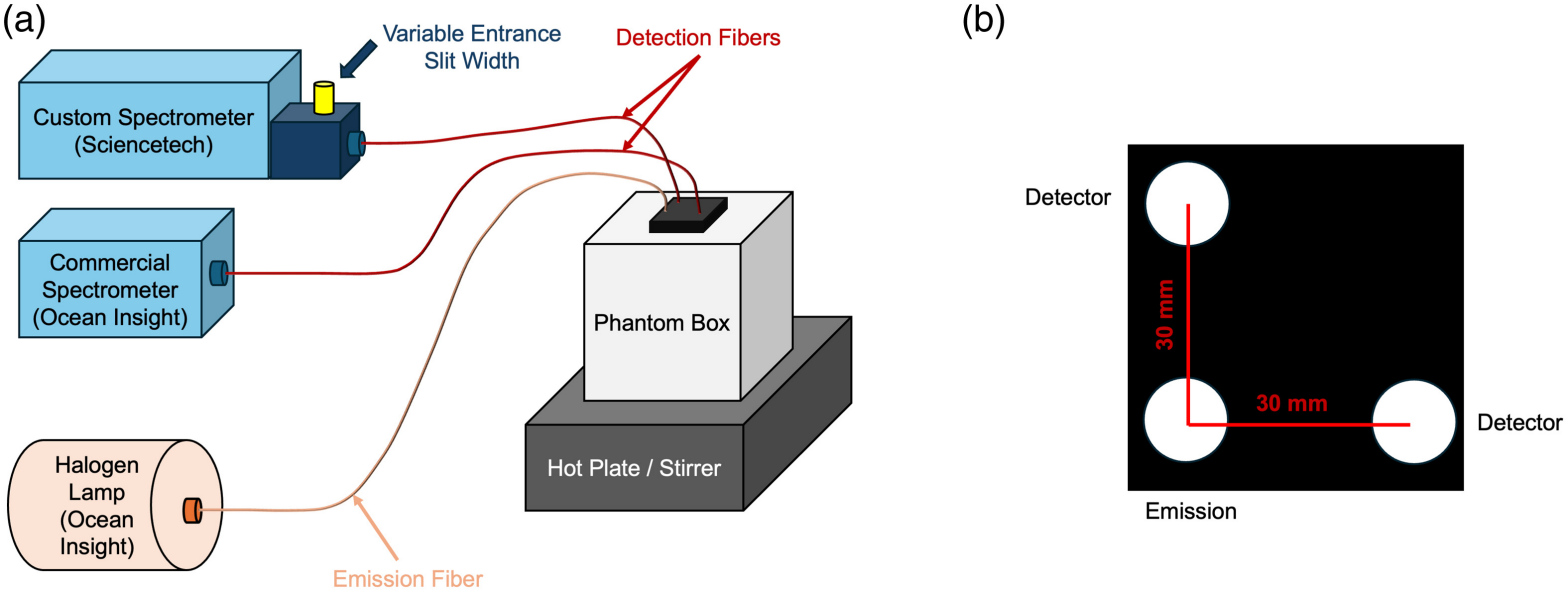
(a) Simplified schematic of setup used in the phantom experiments (not drawn to scale). The detection fiber probes are shown in red attached to the custom spectrometer with the variable entrance slit as well as an off-the-shelf spectrometer with a fixed slit width. The emission probe is shown in orange attached to the halogen lamp. (b) Magnified representation of the probe holder used in the phantom study, with source-detector separation distances provided in millimeters.

Measurements were acquired in blood-lipid phantoms in either an oxygenated or deoxygenated state. The composition of the phantoms was based on previous reports.^29^^–^^34^ Briefly, each phantom was made using a 0.8% Intralipid solution, prepared in an opaque polyvinyl chloride (PVC) container (20×20×20  cm, L×W×H) by diluting 168 mL of Intralipid-20% with 4008 mL of distilled water. Twenty-four milliliters of sodium-bicarbonate buffer were added to maintain physiological pH (7.4) during deoxygenation. The phantom was placed on a hot plate to heat the solution to typical human body temperature (36°C to 37°C). Homogeneity of the solution was maintained throughout the experiment with a magnetic stir bar (Fisherbrand Isotemp Hot Plate Stirrer, Temecula, California, United States). The tips of the optical probes, which were connected to the halogen lamp for emission and the spectrometer for detection, were placed at the center of the phantom 30 mm apart and were slightly submerged to maintain good contact with the liquid as the phantom was stirred (Fig. 1).^30^

Next, 10 mL of whole canine blood was added to each phantom. The liquid was then stirred uncovered for 15 min to allow for gas exchange at the surface of the phantom to stabilize before the measurements were acquired. Stability was confirmed using continuous spectrum measurements with a commercial spectrometer (Maya2000 Pro, Ocean Insight, Orlando, Florida, United States). Measurements were then acquired from the oxygenated blood phantom at each resolution using the laboratory-grade spectrometer by varying the entrance slit.

Thereafter, the phantoms were fully deoxygenated by adding 8 g of baker’s yeast dissolved in 8 mL of a 50% glucose solution and 4 mL of sodium-bicarbonate buffer. Plastic wrap was used to seal the top of the phantom to minimize gas exchange at the surface.^30^ The liquid was stirred for 25 min, and the deoxygenation plateau was confirmed using continuous spectrum measurements from the commercial spectrometer. Measurements were then acquired from the deoxygenated blood phantom at each resolution.

Ten spectra were acquired at each resolution and averaged to increase SNR. Furthermore, the integration time was adjusted to obtain a similar SNR for all resolutions while keeping the intensity of the light source constant. Integration times ranged from 100 ms at the coarsest resolution (19.9 nm) to 30,000 ms at the finest resolution (0.9 nm). Four distinct phantom experiments were conducted on different days to demonstrate repeatability, and the estimated Hb and HbO concentrations were averaged across the four trials.

### 2.3 Simulations

**Fig. 2:**
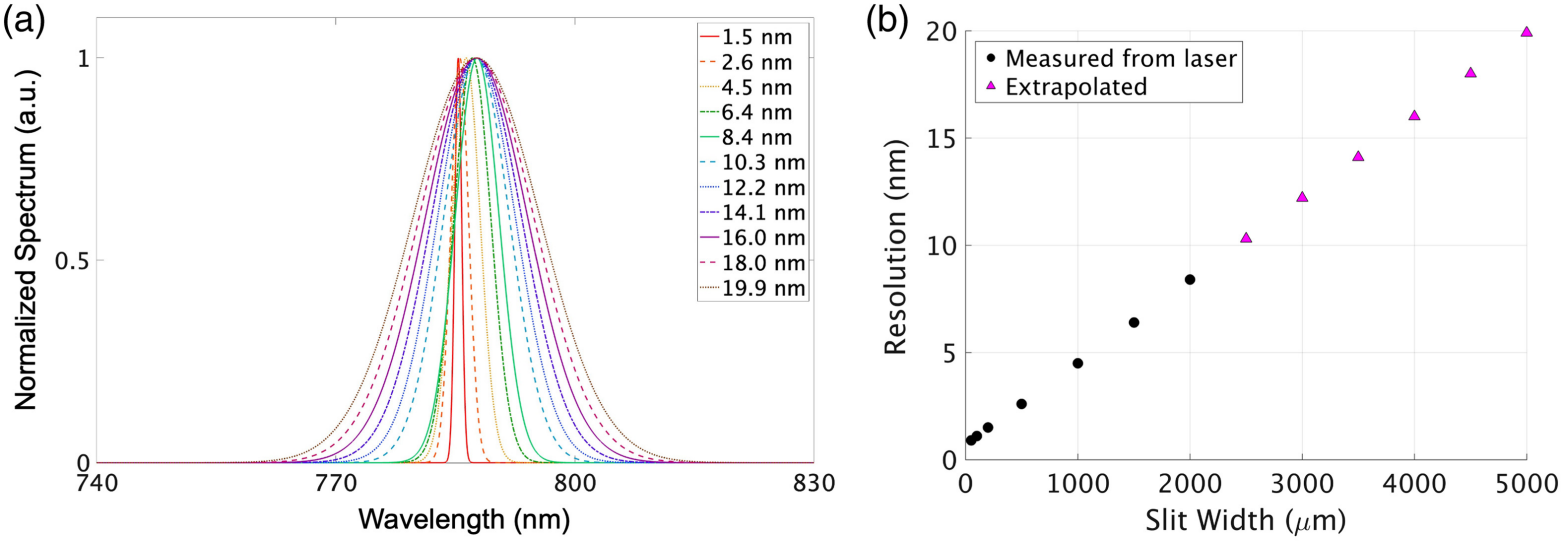
(a) Normalized spectra of the 785-nm laser at different spectral resolutions. (b) Spectral resolution (nanometer) as a function of the width (micrometer) of the spectrometer’s entrance slit. Black dots represent slit widths for which PDFs were measured directly from the laser linewidth, whereas the magenta triangles represent the slit widths for which PDFs were reconstructed from extrapolation of the FWHM.

Spectral pulses from the 785-nm laser were measured using the same spectrometer at slit widths corresponding to 0.9 to 8.4 nm resolutions (Table 1). At wider slit widths (i.e., coarser resolutions), the experimental setup could not attenuate the laser power enough to avoid detector saturation. However, as the FWHM of the laser linewidth had a linear relationship with resolution, the linewidths for the remaining resolutions were simulated by extrapolating the FWHM to the 19.9-nm resolution in step sizes reported in Table 1, thereby capturing the detection-induced spectral broadening of the laser linewidth for all resolutions investigated in the phantom experiments. The spectral pulses were normalized by their area under the curve to convert them into photon probability density functions (PDFs), and Gaussian functions were subsequently fitted to each of the 13 PDFs [Fig. 2(a)].

Mesh-based NIRFAST simulations,^35^^,^^36^ which are based on the diffusion approximation of photon propagation in turbid media, were performed to obtain reflectance spectra at intermediate oxygenation levels not investigated in the phantom study. To mimic the phantom experiments, a 20×20×10  cm mesh was created in 3D Slicer with source and detector fiducials placed 3 cm apart on the surface of the mesh. For each simulation, homogenous optical properties were defined for the mesh with the reduced scattering coefficient set using a power law; the amplitude was set to 0.254 (${\mathrm{mm}}^{-1}$), and the power was 1.7. Note that these parameters represent the average values derived from the four sets of phantom experiments described in Sec. 2.2

$$\mu_{s}^{\prime }(\lambda )=0.254(\lambda /800{)}^{-1.7}.$$ (1)

Furthermore, the absorption coefficient in the simulation was based on the contribution of Hb, HbO, and water, with water fixed at 99% for all simulations, whereas Hb and HbO concentrations were adjusted such that the oxygen saturations vary from 40% to 90% in increments of 10% [Eq. (2)]. Literature extinction coefficients (in ${\mathrm{mm}}^{-1}\mu m^{-1}$) used for the chromophores had a resolution of 1 nm and spanned 650 to 898 nm^37^

$$\mu_{a}(\lambda )=[\mathrm{Hb}]\varepsilon_{\mathrm{Hb}}(\lambda )+[\mathrm{HbO}]\varepsilon_{\mathrm{HbO}}(\lambda )+[H_{2}O]\varepsilon_{H_{2}O}(\lambda ).$$ (2)

Note that the refractive index was set to that of water (i.e., 1.33) for all simulations.

The simulated attenuation spectra for all oxygen saturations were convolved with 11 of the 13 Gaussian functions, omitting the first two slit widths as they were approximately equal to or finer than the 1.0-nm resolution of the literature extinction coefficients used for the simulations.^37^ The convolution was performed to mimic the detection-induced spectral broadening at each resolution^38^

$$A_{i}(\lambda )=A_{0}(\lambda )*{\mathrm{PDF}}_{i},$$ (3)

where $A_{0}(\lambda )$ is the simulated attenuation spectrum, ${\mathrm{PDF}}_{i}$ is the probability density function at resolution i, and $A_{i}(\lambda )$ is the attenuation spectrum at resolution i. There was a slight shift in the position of the laser peak as the slit width was increased [Fig. 2(a)]. To test the effects of this shift, Gaussian functions centered at varying wavelengths other than 785 nm (i.e., ±100  nm) were tested, and all yielded similar spectral attenuation. Experimental noise measured from five separate blood-lipid phantom experiments, using the same optical setup, was added to each simulation to mimic five repetitions, each with a unique noise level. For each phantom, the noise spectrum was quantified by applying a wavelet-based NIRS denoising pipeline to the experimental reflectance.^10^ Thereafter, the denoised spectrum was subtracted from the measured spectrum to obtain the noise spectrum, which was normalized by the denoised spectrum to obtain 1/SNR. For each simulation, 1/SNR was multiplied by the simulated clean NIRFAST spectrum to obtain a noise spectrum, which was then added to the simulated spectrum. The mean SNRs across the wavelength range were as follows for the five different repetitions: 1051, 1290, 1554, 1942, and 32,561.

### 2.4 Data Processing and Analysis

Chromophore concentrations in the phantom and simulation data were estimated by differential spectral analysis based on the solution to the diffusion equation for a semi-infinite homogeneous medium.^6^^,^^7^^,^^10^^,^^39^ In the phantom experiments, HbO concentration was estimated from the oxygenated blood phantoms, whereas Hb concentration was obtained from the deoxygenated phantoms. For the simulations, both chromophores were quantified at each saturation between 40% and 90%. HbO concentration was recovered using the first derivative of the attenuation spectrum between 690 and 850 nm, whereas Hb concentration was estimated using the second derivative of the spectrum between 700 and 800 nm. The water fraction was assumed to remain constant at 99%.

To provide a ground truth for the phantom experiments, the total hemoglobin concentration (g/dL) in each phantom was computed as one-third of the measured packed cell volume of blood used in the phantom.^40^^,^^41^ Subsequent unit conversion to μM was determined using a molecular weight of 64,500 g/mol for hemoglobin. For the simulations, the input values for Hb and HbO served as ground truth.

For the experimental data, the absolute difference between the ground truth total hemoglobin concentrations and the recovered concentrations of HbO (in the fully oxygenated phantom) and Hb (deoxygenated phantom) was computed at each resolution. This difference, expressed in micromolar, was assumed to be the absolute error in the estimated chromophore concentration in the phantom due to changes in spectral resolution. For the simulations, errors in the estimation of the chromophores at the different resolutions were quantified as the difference (in micromolar) between the input chromophore concentrations and the estimated values obtained by fitting the simulated attenuation spectra. For easier interpretation, the errors were expressed as a percentage of the total hemoglobin present in the phantoms or simulations.

Normality in the errors of the estimated concentration of each chromophore was confirmed through a visual inspection of a quantile–quantile (QQ) plot. To categorize the resolutions, k-means clustering was applied to the phantom and simulation data separately based on the errors in chromophore concentration estimates across different resolutions. An elbow test was performed to determine the optimal number of clusters. Subsequently, a one-way analysis of variance (ANOVA) was used to compare the errors in the estimated concentrations of Hb and HbO across the different resolution clusters.

## 3 Results

### 3.1 Experimental Results

**Fig. 3:**
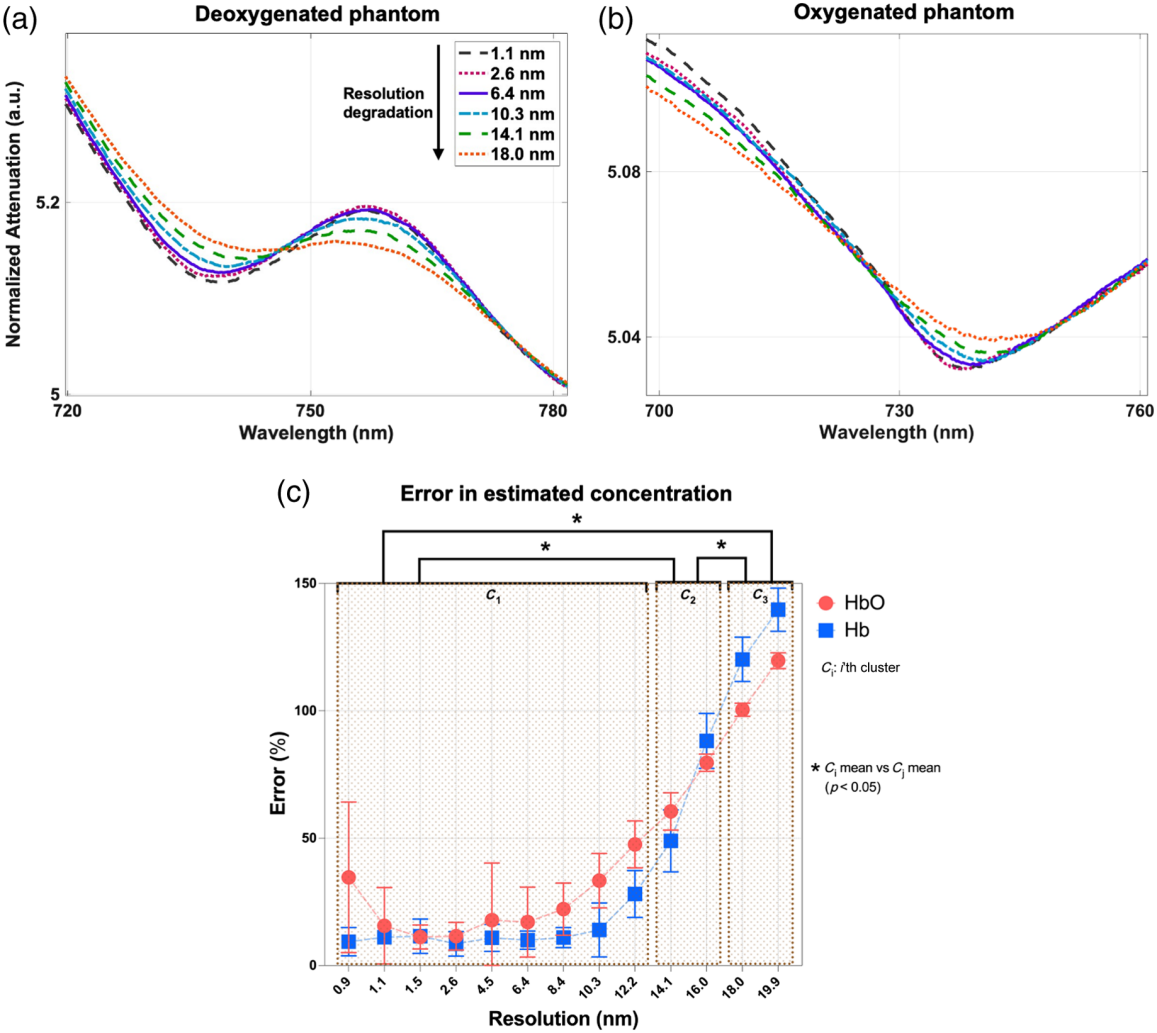
Normalized attenuation spectra of hemoglobin for (a) a fully deoxygenated and (b) a fully oxygenated phantom at six resolutions. (c) Error (±SD) in the estimated chromophore concentration for each resolution with respect to the hemoglobin concentration obtained from the packed cell volume, with each bracket representing a different resolution cluster. Asterisks indicate that the mean errors among clusters are significantly different (one-way ANOVA; p<0.05).

Figures 3(a) and 3(b) depict examples of spectra measured on deoxygenated and oxygenated blood-lipid phantoms at six spectral resolutions and illustrate the typical detection-induced spectral broadening observed at coarser resolutions. Figure 3(c) displays the errors in the estimated chromophore concentration for each resolution with respect to the hemoglobin concentration obtained from the packed cell volume (i.e., the ground truth). The errors in the estimated Hb and HbO concentrations could be objectively divided into three resolution groups using k-means clustering: 0.9 to 12.2 nm (cluster 1), 14.1 to 16.0 nm (cluster 2), and 18.0 to 19.9 nm (cluster 3). In cluster 1, the mean errors between the estimated hemoglobin concentrations and the ground truth were 12.7±7.9% for Hb and 23.4 ± 18.0% for HbO. Furthermore, the errors in the estimated concentration of both chromophores steadily increased with slit width for spectral resolutions beyond 12.2 nm. Notably, the errors in the estimated chromophore concentrations significantly increased in the other clusters for both Hb (p<0.05) and HbO (p<0.05).

### 3.2 Simulation Results

**Fig. 4:**
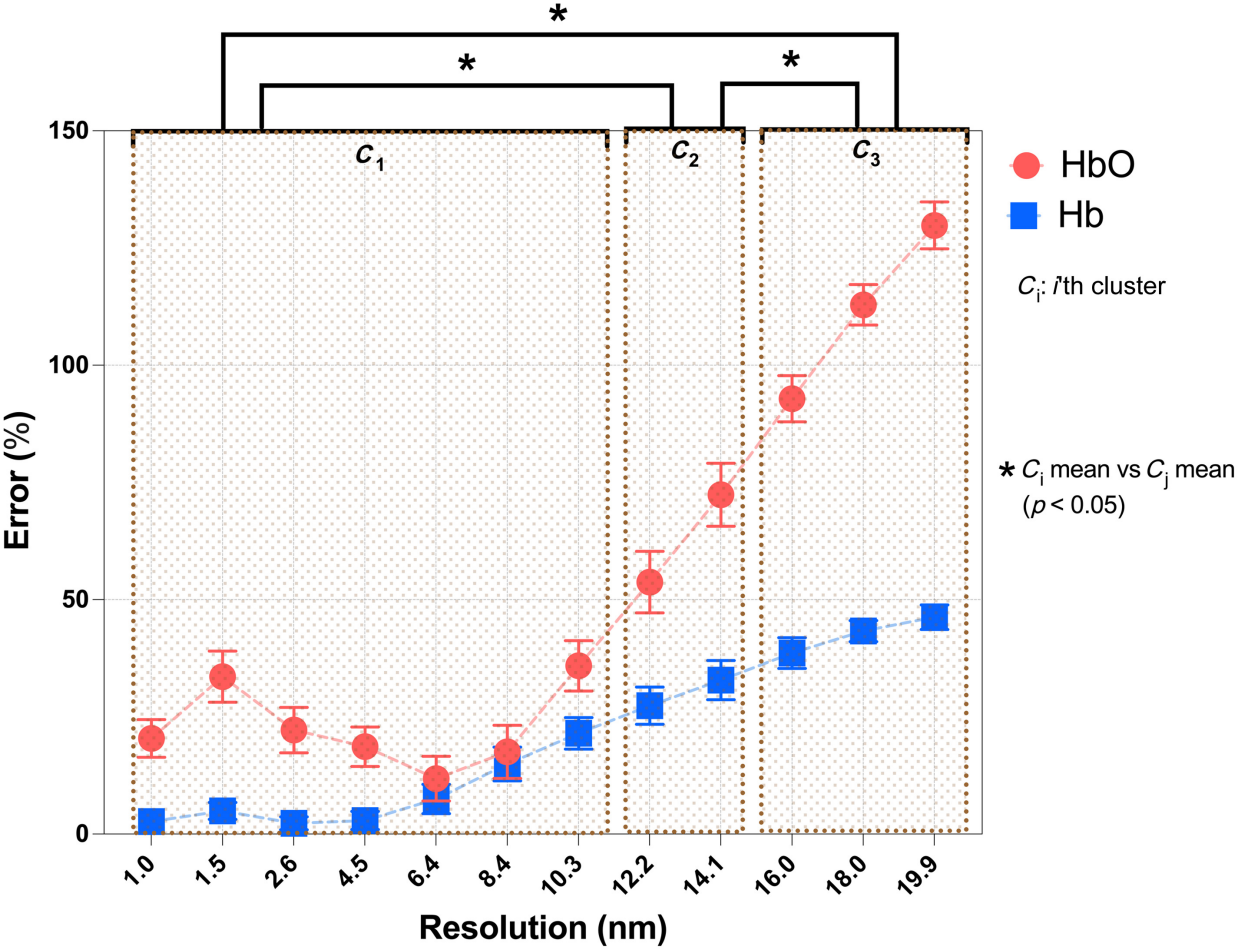
Error (±SD) in the estimated chromophore concentration for each resolution with respect to the ground truth, with each bracket representing a different resolution cluster. Asterisks indicate that the mean errors among clusters are significantly different (one-way ANOVA; p<0.05).

Figure 4 displays the errors in the estimated chromophore concentration for each resolution with respect to the simulated hemoglobin concentration (i.e., the ground truth). Similar to the phantom experiments, the errors in the estimated Hb and HbO concentrations could be objectively divided into three resolution groups using k-means clustering: 1.0 to 10.3 nm (cluster 1), 12.2 to 14.1 nm (cluster 2), and 16.0 to 19.9 nm (cluster 3). In cluster 1, the mean errors between the estimated hemoglobin concentrations and the ground truth were 8.1±7.3% for Hb and 22.9±9.5% for HbO. The errors in the estimated chromophore concentrations significantly increased in the other clusters for both Hb (p<0.05) and HbO (p<0.05).

## 4 Discussion

Continuous-wave NIRS (cwNIRS) is an attractive alternative to frequency-domain and time-domain NIRS for *in vivo* tissue spectroscopy. However, current cwNIRS tissue oximeters are either equipped with only a few discrete wavelengths, which restricts them to only measuring relative changes in tissue chromophore concentrations, or are based on expensive custom-built spectrometers. The development of low-cost, off-the-shelf spectrometers with high SNR could significantly increase the clinical adoption of cwNIRS. Nevertheless, the coarsest spectral resolution that maximizes SNR while preserving accuracy has not yet been established for hyperspectral cwNIRS (h-NIRS) tissue oximetry.

In this work, the minimum spectral resolution required for h-NIRS tissue oximetry was determined by evaluating the accuracy of HbO and Hb estimations at several resolutions in tissue-mimicking blood-lipid phantoms and virtual phantoms (i.e., simulations). The analysis revealed that the estimated concentrations of HbO and Hb are relatively consistent up to a resolution of 10.3 nm. For the phantom experiments, the last resolution of cluster 1 was 12.2 nm; however, 10.3 nm was the largest mutual resolution in the first cluster for both the phantom experiments and simulations [Figs. 3(c) and 4]. Notably, for all spectral resolutions up to 10.3 nm, the errors of Hb estimates were consistently low (i.e., 10.8±5.4% for phantom experiments and 8.1±7.3% for simulations), after which a steady and significant increase was observed. The errors of HbO estimates were slightly higher (i.e., 20.4±16.6% for phantom experiments and 22.9±9.5% for simulations), but a steady increase in error, similar to the Hb estimates, was observed above 10.3 nm. The lower accuracy in the HbO estimates is likely due to crosstalk with light scattering as the effects of scattering are stronger in the first than the second derivative.^7^ Importantly, these results suggest that a resolution as coarse as 10 nm can be used to boost SNR while still providing accurate estimates of Hb and HbO. This resolution is considerably coarser than the range of spectral resolutions (0.3 to 5.5 nm) reported in the literature.^10^^,^^12^^,^^20^^,^^23^^–^^26^^,^^28^ Furthermore, based on the spectra in Figs. 3(a) and 3(b), we speculate that if the extinction coefficients of oxygenated and deoxygenated blood were measured at coarser resolution and used to analyze attenuation spectra acquired with a h-NIRS device with a similar resolution, the errors might be smaller; however, this hypothesis needs to be tested.

In the phantom study, measurements were acquired only from fully oxygenated and fully deoxygenated phantoms, without taking measurements at intermediate oxygenation levels. This is because the presence of yeast in the phantoms made it challenging to maintain a stable intermediate oxygenation over a prolonged period to acquire attenuation spectra at different resolutions. To mitigate this limitation, spectra for intermediate oxygenation levels were simulated using NIRFAST. The errors in the estimated chromophore concentrations from the phantom experiments and simulations yielded similar clusters and trends; however, there are some differences that are likely due to experimental limitations: (1) When the phantom is fully deoxygenated with the addition of yeast, the Hb concentration should be equal to the total hemoglobin estimated from the packed cell volume; however, due to experimental limitations, it is difficult to truly achieve 100% blood deoxygenation, leading to larger errors in the estimated Hb concentrations in the phantom, compared with the simulations where the desired oxygen saturations are truly achieved. (2) The simulations had a perfect ground truth as the input concentrations of Hb and HbO for the simulations are known. However, the ground truth of the phantom study was based on a total hemoglobin concentration in g/dL estimated as one-third of the packed cell volume of blood. As such, the phantom data include potential errors in the estimated packed cell volume and method of conversion to hemoglobin concentration, as well as any potential biases introduced by the h-NIRS hardware. (3) In the phantom study, measurements were only acquired at full deoxygenation and full oxygenation. In these extreme states, light absorption due to blood is dominated by only one of the two hemoglobin chromophores (Hb in full deoxygenation and HbO in full oxygenation). As such, there was minimal crosstalk in the estimation of the chromophores in these two states. The simulations, however, were conducted at nonextreme oxygen saturation levels (i.e., 40% to 90%), when both chromophores have significant contributions to the measurements, potentially leading to crosstalk.

Oxygen saturation in the phantoms and simulations was also computed as the percentage ratio of oxygenated hemoglobin concentration to the total hemoglobin concentration. However, cluster analysis did not reveal any resolution-dependent clusters as the errors did not significantly vary with resolution. One explanation for this is that when similar error factors are present in the estimated concentrations of HbO and Hb, one would erroneously arrive at accurate oxygenation estimates, whereas the absolute hemoglobin concentrations are inaccurate.

Furthermore, as water absorption is weak in the spectral range used in this study (600 to 900 nm), changes in its concentration are expected to have minimal effects on the accuracy of the estimation of the hemoglobin concentrations. Notably, *in vivo* studies have shown that a 10% error in the estimated water concentration has small effects on the accuracy of the estimated tissue blood content and oxygenation (only 2% to 3% error).^42^ However, an increase in hemoglobin concentration could lead to more absorption and consequently lower SNR, which would result in lower accuracy in the estimated hemoglobin concentration. It is noteworthy that this should not be an issue if the hemoglobin content is similar to *in vivo* concentrations. By contrast, a decreased hemoglobin concentration should lead to increased SNR and a positive effect, especially on the finest spectral resolution, for which the very narrow slit width leads to lower light collection. Given that hemoglobin is a very strong light absorber in the spectral range of interest, its concentration must be reduced to traces for low concentration to become an issue. We further hypothesize that the presence of myoglobin in tissue (e.g., muscle) would not impact the resolution required to resolve the absorption features of the chromophores as the spectral shape of myoglobin absorption and that of its derivatives are nearly identical to those of hemoglobin.^43^

A major advantage of hyperspectral NIRS is that it allows for the use of spectral derivatives, which provide several benefits compared with other inverse methods. These advantages include high immunity to movement artifacts and changes in the coupling between the optical probes and the tissue.^7^ Another commonly used technique for analyzing hyperspectral NIRS data is the Beer–Lambert method. In this approach, the optical pathlength in the medium is either estimated or, more commonly, assumed.^9^^,^^44^ When the optical pathlength is estimated, we anticipate the results of the Beer–Lambert analysis to closely align with those reported in the current study. However, if the optical pathlength is assumed, any inaccuracies in the assumption will directly impact the estimated hemoglobin concentrations, potentially introducing errors. It is essential to note that changes in tissue absorption cause alterations of the optical pathlength, and these alterations are wavelength dependent. Therefore, any h-NIRS analysis method should appropriately account for this potential confounder to ensure accurate estimation of Hb and HbO concentrations.

The homogeneous phantoms and simulations used in this study do not reflect the structural complexity that is typically encountered *in vivo*. For instance, in noninvasive optical neuromonitoring, light must go through the scalp and skull before reaching the brain. However, using structurally complex phantoms could introduce additional confounders and make interpretation of the results more challenging. Notably, it would be difficult to determine if errors in the estimated hemoglobin content were due to the confounding effect of the phantom structure (e.g., the presence of extra layers) or changes in spectral resolution.

## 5 Conclusion

In this study, we determined that the minimal spectral resolution for h-NIRS tissue oximetry is 10 nm, a resolution that is considerably coarser than the typical values reported in the literature (0.3 to 5.5 nm).^10^^,^^12^^,^^20^^,^^23^^–^^26^^,^^28^ These results suggest that a coarser resolution could be used without impacting the accuracy of the estimation of tissue blood content and oxygenation. Furthermore, the increased light throughput afforded by a wider slit width could allow for shorter integration times and higher SNR. Ultimately, the implementation of this optimal resolution in compact, off-the-shelf spectrometers could increase the adaption of this technology for low-cost, quantitative point-of-care tissue monitoring techniques.

## Data Availability

Code and data that support the findings of this study are available from the corresponding author upon reasonable request.
